# Application of array comparative genomic hybridization in 256 patients with developmental delay or intellectual disability

**DOI:** 10.1007/s13353-013-0181-x

**Published:** 2013-12-03

**Authors:** Magdalena Bartnik, Beata Nowakowska, Katarzyna Derwińska, Barbara Wiśniowiecka-Kowalnik, Marta Kędzior, Joanna Bernaciak, Kamila Ziemkiewicz, Tomasz Gambin, Maciej Sykulski, Natalia Bezniakow, Lech Korniszewski, Anna Kutkowska-Kaźmierczak, Jakub Klapecki, Krzysztof Szczałuba, Chad A. Shaw, Tadeusz Mazurczak, Anna Gambin, Ewa Obersztyn, Ewa Bocian, Paweł Stankiewicz

**Affiliations:** 1 Department of Medical Genetics, Institute of Mother and Child, Warsaw, Poland; 2 Institute of Computer Science, Warsaw University of Technology, Warsaw, Poland; 3 Institute of Informatics, University of Warsaw, Warsaw, Poland; 4 Genetic Counseling Unit, Institute of Physiology and Pathology of Hearing, World Hearing Center, Warsaw, Poland; 5 Department of Molecular and Human Genetics, Baylor College of Medicine, One Baylor Plaza, Rm. R809, Houston, TX 77030 USA; 6 Mossakowski Medical Research Centre, Polish Academy of Sciences, Warsaw, Poland

**Keywords:** Copy-number variation, Microdeletion, Microduplication, Chromosomal microarray analysis

## Abstract

We used whole-genome exon-targeted oligonucleotide array comparative genomic hybridization (array CGH) in a cohort of 256 patients with developmental delay (DD)/intellectual disability (ID) with or without dysmorphic features, additional neurodevelopmental abnormalities, and/or congenital malformations. In 69 patients, we identified 84 non-polymorphic copy-number variants, among which 41 are known to be clinically relevant, including two recently described deletions, 4q21.21q21.22 and 17q24.2. Chromosomal microarray analysis revealed also 15 potentially pathogenic changes, including three rare deletions, 5q35.3, 10q21.3, and 13q12.11. Additionally, we found 28 copy-number variants of unknown clinical significance. Our results further support the notion that copy-number variants significantly contribute to the genetic etiology of DD/ID and emphasize the efficacy of the detection of novel candidate genes for neurodevelopmental disorders by whole-genome array CGH.

**Electronic supplementary material:**

The online version of this article (doi:10.1007/s13353-013-0181-x) contains supplementary material, which is available to authorized users.

## Introduction

Developmental delay (DD)/intellectual disability (ID), characterized by a significant impairment of cognitive and adaptive functions, affects 1–3 % of the general population (Harris 2006; Maulik et al. 2011), with the majority of affected individuals remaining without proper diagnosis (Rauch et al. 2006; Pfundt and Veltman 2012). The etiology of DD/ID is heterogeneous, with both genetic and environmental contribution (Grayton et al. 2012). In addition to Mendelian DD/ID, one of the most common causes are microscopically visible chromosomal aberrations and submicroscopic copy-number variants (CNVs) (Morrow 2010; Regier et al. 2010). A high-resolution G-banded karyotype reveals chromosome abnormalities in 3–5 % of patients with idiopathic DD/ID (Shevell et al. 2003), and molecular cytogenetic analyses, e.g., fluorescent in situ hybridization (FISH) in the subtelomeric regions, provides diagnosis in an additional 3–6 % of cases (Koolen et al. 2004; Ravnan et al. 2006). In the past few years, application of chromosomal microarray analysis (CMA), including array comparative genomic hybridization (array CGH) and single-nucleotide polymorphism (SNP) arrays, has revolutionized the clinical diagnostics in patients with idiopathic DD/ID (Cooper et al. 2011; Kaminsky et al. 2011; Girirajan et al. 2012). Recently, it has been proven that CMA should be a first-tier clinical diagnostic test for individuals with DD, ID, autism spectrum disorders, and dysmorphic features (Miller et al. 2010; Battaglia et al. 2013). Clinically relevant CNVs, ranging in size from megabases to kilobases (Rodriguez-Revenga et al. 2007), have been detected in ∼10–20 % of cases (Menten et al. 2006; Stankiewicz and Beaudet 2007; Koolen et al. 2009) and have led to the identification of several novel microdeletions and microduplications associated with DD/ID (Slavotinek 2008; Vissers and Stankiewicz 2012), e.g., involving chromosomes 1q41q42, 9q22.3, 15q13.3, 15q24, 16p11.2, and 17q21.31 (Koolen et al. 2006; Redon et al. 2006; Sharp et al. 2006; Shaw-Smith et al. 2006; Ballif et al. 2007; Shaffer et al. 2007; Sharp et al. 2007, 2008). Refinement of the critical region of a known syndrome by the identification of atypical deletion (Cooper et al. 2011) may facilitate the detection of a dosage-sensitive gene(s) related to ID (Vissers et al. 2010); however, there are many CNVs for which the clinical significance may still remain unknown (Rodriguez-Revenga et al. 2007). Recently, clinical and biological interpretation of those variants and their genotype–phenotype correlation enabled the generation of a human genome morbid map (Cooper et al. 2011).

Here, we present the results of the application of genome-wide array CGH in a cohort of 256 patients with DD/ID, dysmorphic features, congenital malformations, or additional neurodevelopmental abnormalities. We identified 84 non-polymorphic CNVs in 69 patients, including 41 clinically relevant CNVs, 15 potentially novel pathogenic genetic loci for DD/ID, and, additionally, 28 CNVs of unknown clinical significance, likely to be non-pathogenic changes.

## Materials and methods

### Patients

We studied 256 patients with DD/ID, dysmorphic features, congenital malformations, or additional neurodevelopmental abnormalities. Among them, 234 patients had normal GTG banding analysis with at least 550-band resolution and 46 patients had a negative fragile X testing.

We provide a detailed clinical description of five patients (Supplementary material) and discuss their genotype–phenotype correlations. Patients 23 and 34 have de novo CNVs that we believe are pathogenic, and patients 45, 50, and 51 have potentially pathogenic CNVs.

### DNA isolation

Genomic DNA was extracted from peripheral blood cells using a Puregene DNA Blood Kit (Qiagen, Gentra Systems, Minneapolis, MN), according to the manufacturer’s protocol. The reference DNA samples were obtained from phenotypically normal male and female controls.

### Array CGH

Custom-designed exon-targeted clinical array CGH was performed using 105K V7.4 and 180K V8.0 or V8.1 microarrays designed in the Medical Genetics Laboratories at Baylor College of Medicine (BCM) (http://www.bcm.edu/geneticlabs/cma/tables.html) in cooperation with the Department of Medical Genetics at the Institute of Mother and Child and manufactured by Agilent Technologies (Santa Clara, CA). V7.4 consisted of 105,000 oligonucleotides having genome-wide coverage with an average resolution of 30 kb, while V8.0 and V8.1 OLIGO (180K) arrays have genome-wide coverage as well as exon coverage for over 1,700 known or candidate disease genes with an average of 4.2 oligos per exon and intronic gaps no larger than 10 kb (Boone et al. 2010). The microarray used in this study does not contain SNP probes and it does not detect regions of absence of heterozygosity (AOH). The results of studies using the updated version of this array were recently published by the BCM (Wiszniewska et al. 2013). Digestion, labeling, and hybridization were performed following the manufacturer’s instructions. The BCM web-based software platform and the homebrew IMiD-web2py software were used for chromosomal microarray analysis. All genomic coordinates are based on the March 2006 assembly of the reference genome (NCBI36/hg18). To verify the rearrangements identified by array CGH, depending on CNVs size, we used GTG banding and FISH. When available, blood samples were obtained from patients’ parents, and CNV inheritance was investigated.

### Karyotype analysis

GTG banding analysis was performed according to the standard protocol in peripheral blood lymphocytes. The metaphases with 550-band resolution were analyzed in cases with CNVs 5 Mb or greater in size.

### Fluorescent in situ hybridization (FISH) analysis

Confirmatory FISH experiments were performed to verify the presence of CNVs ranging in size from 150 kb to 5 Mb. FISH analyses were performed by standard procedures in phytohemagglutinin-stimulated peripheral blood lymphocytes using probes derived from bacterial artificial chromosomes. When available, blood samples were obtained from the patient’s parents and the origin of the identified CNVs was studied using FISH with the same probes.

## Results

A total of 84 non-polymorphic CNVs were found in 69 of 256 patients studied. We divided the detected CNVs into three groups. The first group contains 41 CNVs considered to be clinically relevant (i.e., pathogenic for DD/ID) (Table 1). This group includes 18 imbalances greater than 5 Mb in size identified in 17 patients (not seen in standard cytogenetic studies) and 23 submicroscopic CNVs. In three cases, two pathogenic CNVs were detected: patient 9 had an unbalanced translocation der(10)t(10;20)(q26.2;q13.33); patient 10 had a terminal duplication dup(11)(p15) and a terminal deletion del(11)(q24), most likely a recombination product of a pericentromeric inversion inv(11)(p15q24); and patient 12 had a large 15q13.3q14 deletion in addition to the well-known recurrent microdeletion 16p13.11. We also identified two mosaic trisomies of chromosome 9 (pt 15 and pt 16) and monosomy of chromosome 7 in a 1-year-old patient (pt 17) with DD and combined immunodeficiency. Among 23 submicroscopic CNVs, we identified known recurrent rearrangements, e.g., deletions 15q11.2, 16p13.3, 17q11.2, and 17q21.31 and different-sized non-recurrent CNVs, e.g., deletions 1p36.31p36.33, 1q43q44, 5q14.3, 6q25, and 10q24.32, as well as duplications 3p21.1 and 19p13.3. In this group, we also identified two recently described microdeletions at chromosomes 4q21 and 17q24.2.

**Table 1:** CNVs clinically relevant for DD/ID

Pt	Sex	Age (years)	Previous negative genetic tests	aCGH results (hg 18)	Best candidate genes	Size (Mb)	Verification/parental studies	Inheritance	Clinical features	MIM or citation
CNVs > 5 Mb										
1^a^	M	2	–	1p31.3p31.1 (64,387,592–76,910,889)x1	*NEGR1*	12.5	Karyotype/karyotype	de novo	Delayed psychomotor development, speech delay, muscular hypotonia; dysmorphic features: down-slanting palpebral fissures, epicanthic folds, telecanthus, broad nasal bridge, downturned corners of the mouth, “whistling” mouth appearance, low-set ears, and protruding ears	(Petti et al. 2011)
2	F	8	Karyotype/FISH (22q11.2)	1q41q43 (221,641,959–241,103,255)x3	*DISP1*, *LBR*, *LEFTY1*, *LEFTY2*, *PSEN2*, *WNT3A*	19.4	–	unknown	Delayed psychomotor development, mild ID (IQ 52), speech delay, learning difficulties, IUGR, failure to thrive, postnatal microcephaly (−2.05 SD), heart defect (ASD, extra left superior vena cava), small hands and feet; dysmorphic features: hypotelorism, synophrys, flat philtrum, prominent nose, micrognathia, low posterior hairline, hypoplastic labia minora, and hirsutism	612530
3	F	13	Karyotype	2q37.2q37.3 (235,388,743–242,329,883)x1	*HDAC4*	6.9	Karyotype/karyotype	de novo	Delayed psychomotor development, profound ID, speech delay (only single words), infantile muscular hypotonia, excessive weight gain, and postnatal obesity; dysmorphic features: low posterior hairline, broad nasal tip, high forehead, “mask” face nystagmus, brachydactyly, single palmar crease, and feet abnormalities	600430
4	F	8	Karyotype	5q14.3q15 (87,135,909–92,540,627)x1	*MEF2C*, *GPR98*	5.4	Karyotype/karyotype	de novo	Delayed psychomotor development severe ID, speech delay, muscular hypotonia, epilepsy, and stereotyped movements of the limbs; mild dysmorphic features: facial asymmetry, flat occiput, strabismus, and congenital cataract; family history: proband’s father’s cousin: profound ID, epilepsy; mother and grandfather: kidney duplication	613443
5	F	5	Karyotype/subtelomeric test/FISH (22q11.2)	5q35.1q35.3 (171,841,772–177,881,782)x1	*NSD1*	6.0	–	unknown	Severe ID (IQ 30), absent speech, muscular hypertonia/axial hypotonia, heart defect (VSD, pulmonary stenosis, PDA), scoliosis, 13 ribs, vesicoureteral reflux, hypothyroidism, macrocephaly, postnatal short stature, and epilepsy; dysmorphic features: high palate, micrognathia, prominent forehead, long face, and macrostomia; Sotos syndrome features	117550
6	M	3	Karyotype	6q16.1q21 (93,375,185–108,241,524)x1	*FUT9*, *GPR63*	14.8	FISH/FISH	de novo	Delayed psychomotor development, severe ID, speech delay (only single words), muscular hypotonia, postnatal microcephaly, laryngomalacia, umbilical/inguinal hernia, syndactyly of the 2nd and 3rd fingers, fetal finger pads, syndactyly of the 3rd and 4th toes, and constipation; dysmorphic features: flat facial profile, up-slanting palpebral fissures, and lacrimal duct atresia	(Derwińska et al. 2009)
7	F	13	Karyotype/subtelomeric test	8q21.11 (74,765,817–80,451,006)x1	*ZFHX4*	5.6	FISH/FISH	de novo	Mild ID, recurrent upper respiratory tract infections, and umbilical hernia; dysmorphic features: hypertelorism, narrow palpebral fissures (ptosis), epicanthic folds telecanthus, frontal bossing with receding hairline, wide nasal bridge short, flat nose, hypoplasia of nasal cartilage, specific upper lip line appearance, short philtrum, low-set ears, micrognathia, malocclusion, single palmar transverse crease, astigmatism, and hypermetropia	614230
8	M	18	Karyotype/FraX	10p14p12.31 (10,595,409–22,213,068)x1	*NEBL*	11.6	Karyotype/karyotype	de novo	Severe ID (IQ 30), epilepsy, moderate deafness, brain MRI: focal cortical dysplasia, strabismus, and pre-/postnatal microcephaly; dysmorphic features: up-slanting palpebral fissures, epicanthic folds, high palate, micrognathia, short philtrum, full lips, and unusual dark skin pigmentation	601362
9*	M	17	Karyotype	10q26.2q26.3 (129,702,903–134,889,739)x1 20q13.33 (61,395,731–62,194,881)x3 *der(10)t(10;20)(q26.2;q13.33)	*SYCE1* *CHRNA4*, *KCNQ2*	5.1 0.8	FISH/FISH	mat mat	Moderate ID, epilepsy, stereotypic limbs movements, poor suck in the neonatal period, and muscular hypotonia; dysmorphic features: up-slanting palpebral fissures, hypotelorism, low posterior hairline, bulbous nasal tip, broad, short neck fetal finger pads, narrowed upper part of the thorax, strabismus, and hypermetropia	609625 613720
10	F	7	Karyotype/microdeletion test/FISH (22q11.2)	11p15.5p15.2 (186,855–14,246,023)x3 11q24.3q25(128,498,532–134,382,529)x1	*KCNQ1*, *H19*, *CDKN1C* *B3GAT1*	14.0 5.8	Karyotype/karyotype	not mat not mat	Profound ID, speech delay (only single words), epilepsy, neonatal absent suck/feeding difficulties, postnatal short stature and growth failure, muscular hypotonia, and cardiac defect (ASD, pulmonary hypertension, and tricuspid valve insufficiency); dysmorphic features: high palate, prognathism, macrostomia, wide-spaced teeth, midface hypoplasia, deep-set eyes, straight eyebrows, and clubbed fingers	130650 147791
11	F	1	Karyotype	12q14.1q21.1 (56,803,356–70,388,372)x1	*LEMD3*	13.5	Karyotype/karyotype	de novo	Delayed psychomotor development, moderate ID, speech delay, stereotypic hand movements, IUGR, and postnatal severe growth retardation, hypotonia, cleft palate, unilateral cleft lip, hypertrophic cardiomyopathy, corpus callosum hypoplasia, ectopic, small left kidney vesicoureteral reflux, anisocoria, and choroidal defect; dysmorphic features: hypertelorism, micrognathia, synophrys, choroid coloboma, malformed auricles, low-set ears, protruding tongue, and sacral dimple, family history: mother: epilepsy, father: ankylosing spondylitis	166700
12	F	12	Karyotype/subtelomeric test/FISH (22q11.2)	15q13.3q14 (29,516,211–37,530,981)x1 16p13.11 (15,429,214–17,711,867)x1	*CHRNA7* *NDE1*, *MYH11*, *ABCC1*, *ABCC6*	8.0 2.2	Karyotype/Karyotype –/FISH	de novo de novo	Delayed psychomotor development, mild ID, speech delay, cleft palate, brachydactyly, heart defect (pulmonary valve stenosis), and vesicoureteral reflux; dysmorphic features: hypotelorism and micrognathia; family history: distant relatives: case of cleft palate, anal atresia, and history of 2 x miscarriages	612001 (de Kovel et al. 2010)
13	M	3	Karyotype	18q22.1q23 (61,400,117–76,103,255)x1	*TSHZ1*	14.7	Karyotype/karyotype	de novo	Delayed psychomotor development, moderate ID, absent speech, hearing loss, autism spectrum behavior, recurrent vomiting and muscular hypertonia, alternating muscular hypotonia in the neonatal/infantile period, cryptorchidism, hypospadias, omphalocele, strabismus, myopia, and astigmatism; dysmorphic features: high palate, telecanthus, epicanthic folds, single palmar creases, and cafe-au-lait spots	601808
14	F	3	Karyotype/subtelomeric test	Xq22.1q22.3 (100,704,966–105,957,618)x1	*PLP1*	5.2	Karyotype/karyotype	de novo	Delayed psychomotor development, absent speech, muscular hypotonia, and behavioral abnormalities (autoaggression, stereotypies of limb movements); dysmorphic features: hypertelorism, deeply set eyes, short philtrum, pectus excavatum, and abnormal dermatoglyphics	312080
15	F	6	Karyotype/subtelomeric test/FISH (22q11.2)	9p24.3q34.3 (1–140,273,252)x2∼3	–	140.2	Karyotype/–	unknown	Delayed psychomotor development, profound ID, absent speech, pleasant personality, IUGR, feeding difficulties/poor weight gain, muscular hypotonia, postnatal short stature and underweight, heart defect (ASD II), joints laxity, atlantooccipital joint congenital anomaly, duplicated pyelocalyceal system, and brain arachnoidal cyst; dysmorphic features: brachycephaly, asymmetric face, asymmetry of the palpebral fissures micrognathia, dysplastic auricles, and short, webbing neck	(Patil et al. 2012)
16	F	1	Karyotype/methylation test	9p24.3q34.3 (1–140,273,252)x2∼3	–	140.2	Karyotype/–	unknown	Delayed psychomotor development, severe ID, absent suck, feeding problems, recurrent vomiting, Pierre Robin sequence, gastroesophageal reflux disease, constipation, postnatal short stature and growth failure, muscular hypotonia, mixed hearing loss, brain MRI: subcortical atrophy in the occipital lobes, heart defect (aortic coarctation CoA), congenital hip dislocation, talipes calcaneovalgus, and optic disc hypoplasia; dysmorphic features: microstomia, “mask” face, short neck, high forehead, strabismus, partial soft palate cleft, micrognathia, and optic disc coloboma, nystagmus	(Patil et al. 2012)
17	M	1	Karyotype	7p22.3q36.3 (1–158,821,424)x1	–	158.8	–	unknown	Delayed psychomotor development, lack of suck, feeding problems, failure to thrive, muscular hypotonia, and hypospadias; mild dysmorphic features: broad nasal bridge, severe congenital immunodeficiency syndrome with immunoglobulin levels deficiency	(Cordoba et al. 2012)
CNVs < 5 Mb										
18	M	5	Karyotype	1p36.33p36.31 (823,964–5,474,922)x1	*GABRD*, *SKI*	4.6	FISH/FISH	de novo	Delayed psychomotor development, speech delay (moderate hearing loss), postnatal microcephaly (−4.60 SD), postnatal short stature (−2.71 SD), muscular hypotonia, febrile seizures, Dandy–Walker syndrome, agenesis of corpus callosum, heart defect: VSD, duodenal atresia, bilateral inguinal hernia, small hands and feet, hypothyroidism, and nystagmus; dysmorphic features: hypotelorism, flat facial profile, deep-set eyes, small palpebral fissures, dysplastic auricles, broad nasal tip, anteverted nares, long, flat philtrum, microstomia, narrow lips, and straight eyebrows	607872
19^b^	M	12	Karyotype/FISH (15q11.2)	1p22.3p22.2 (86,154,613–90,493,799)x3	*GTF2B*	4.3	FISH/FISH	pat	Moderate ID, delayed speech development (only single words), poor suck and feeding difficulties in neonatal/infancy period, muscular hypotonia, and abnormal brain MRI (cerebellar agenesis, arachnoidal cyst); dysmorphic features: hypertelorism, down-slanting palpebral fissures, broad nasal bridge, bulbous nasal tip, high forehead, tapering fingers, and brachydactyly of the 2nd to 5th toes	–
20	M	7	–	1q21.1 (145,085,612–145,845,306)x1	*GJA5*	0.75	FISH/FISH	de novo	Delayed psychomotor development, absent speech, profound ID, muscular hypotonia, no availability to walking, postnatal microcephaly (−5.82 SD), short stature (−4.43 SD) underweight (−2.77 SD), cryptorchidism, and microcytic hypochromic anemia; dysmorphic features: telecanthus, epicanthic folds, midface hypoplasia, low-set ears, posteriorly rotated ears, flat nasal bridge, macrostomia, tented upper lip, everted lower lip, neonatal feeding difficulties, poor suck, “coarse “ face, open mouth, hypersalivation, molecular confirmation of clinical diagnosis of ATRX syndrome, family history: younger brother also affected with ATRX syndrome but without del1q21.1	612474
21	F	1	Karyotype/subtelomeric test	1q43q44 (239,823,807–243,139,508)x1	*AKT3*	3.3	FISH/FISH	de novo	Delayed psychomotor development profound ID, absent speech (inarticulate sounds), poor suck, failure to thrive in the neonatal and infancy period, muscular hypotonia, postnatal microcephaly (−5.25 SD), epilepsy, abnormal brain MRI: frontal lobe atrophy and corpus callosum hypoplasia, heart defect (VSD), and brachydactyly (2nd toes); dysmorphic features: hypertelorism, low-set ears, flat nasal bridge, preauricular tags (left ear), narrow palpebral fissures, prominent supraorbital ridge, and thick gums	612337
22	M	13	Karyotype/FraX	3p21.1 (51,730,109–54,128,641)x3	*TNNC1*	2.4	FISH/aCGH	de novo	Profound ID, autism spectrum symptoms, absent speech, behavioral abnormalities, (hyperactivity, stereotyped movements), and epilepsy; family history: one case of non-syndromic mild ID in father’s granduncle	–
23	F	8	Karyotype/FISH (15q11.2)	4q21.21q21.22 (81,145,736–84,268,969)x1	*PRKG2*, *RASGEF1B*	3.1	FISH/FISH	de novo	Supplementary material	613509
24	M	2	Karyotype	5q14.3 (88,121,748–88,232,276)x1	*MEF2C*	0.11	FISH/FISH	de novo	Delayed psychomotor development, speech delay (only single words), epilepsy, autism-like hyperactivity behavior, muscular hypotonia and mirror upper limbs movements in the infancy; dysmorphic features: “coarse” facial appearance, high forehead, frontal bossing, epicanthic folds, low-set ears, broad nasal bridge, open mouth, and receding hairline in parietal regions	(Nowakowska et al. 2010)
25	M	7	Karyotype/subtelomeric test/FraX	5q14.3q15 (89,690,573–94,582,832)x1	*GPR98*	4.8	FISH/FISH	de novo	Delayed psychomotor development, mild ID (IQ 68), speech delay, muscular hypertonia, epilepsy; dysmorphic features: protruding ears, frontal bossing	613443
26^c^	M	5	Karyotype/subtelomeric test	6q25.1q25.3 (152,487,219–157,341,421)x1	*ARID1B*	4.8	FISH/aCGH	de novo	Delayed psychomotor development, severe ID, absent speech, inarticulate, single sounds, heart defect: PFO, and hypothyroidism; dysmorphic features: trigonocephaly, thick eyebrows, long eyelashes, epicanthic folds anteverted nares, prominent alae nasi, long, flat philtrum, thin upper lip, open mouth appearance, protruding tongue, protruding ears, pectus excavatum, square distal finger tips, abnormal palmar crease, left cryptorchidism, left inguinal hernia, hypertrichosis, and fair skin	612863
27	M	17	–	10q24.32 (103,852,046–104,127,504)x1	*PITX3*	0.27	PCR	not mat	Mild ID (IQ 65), speech development delay, significant behavior abnormalities (abnormal interpersonal relations, aggressiveness, hyperactivity), self-destructive behavior (onychotillomania, trichotillomania, polyembolokoilamania),, sleep disturbances, seizures episode (abnormal EEG), recurrent respiratory tract infections (until 4 years of age), and idiopathic thrombocytopenia (at the age of 10 years); dysmorphic features: long “coarse” face, high forehead, open mouth, synophrys, short, broad nose, thick, everted lower lip, large ear lobule, thickened helix, brachydactyly type A3, hyperextensible interphalangeal joints, clinodactyly of the IV and V fingers, walking on the external borders of both feet, pes planus, family history: father: learning difficulties, proband’s sister: mild ID (she has a developmentally delayed son)	(Derwińska et al. 2012)
28	F	7	Karyotype/subtelomeric test/BAC aCGH	15q11.2 (20,393,584–20,638,134)x1	*CYFIP1*, *NIPA2*, *NIPA1*	0.24	FISH/FISH	pat	Profound ID, absent speech, and epilepsy; dysmorphic features: prominent supraorbital ridges, smooth, long philtrum, prominent nose, and clinodactyly of the 5th fingers; family history: father: healthy	(Burnside et al. 2011)
29	F	11	Karyotype	16p13.3 (49,978–1,676,053)x1	*HBA2*, *HBA1*	1.6	FISH/FISH	de novo	Moderate ID, speech delay, abnormal brain MRI: enlarged cisterna magna, parietal cortical atrophy, multiple nevi, freckles, and capillary hemangioma on the scalp; mild dysmorphic features: facial symmetry and abnormal philtrum	141750
30^d^	F	6	Karyotype	16p11.2 (29,586,068–30,036,185)x1	*KCTD13*	0.45	FISH/aCGH	pat	Delayed psychomotor development, speech delay, and moderate ID; family history: mother: moderate ID, father: mild ID	611913
31	F	4	Karyotype	17q11.2 (26,133,859–27,245,792)x1	*NF1*	1.1	FISH/FISH	de novo	Delayed psychomotor development, moderate ID, speech delay, hoarse voice, muscular hypotonia, brain MRI: agenesis of corpus callosum, bowed tibia with pseudarthrosis, fetal finger pads, edema of hands, dark skin pigmentation, multiple cafe-au-lait spots and freckles; dysmorphic features: dolichocephaly, coarse face, telecanthus, epicanthic folds, hypertelorism, macrostomia, prominent upper lip/everted lower lip, broad nose, and thick helices strabismus, clinical diagnosis of NF1	613675
32	F	33	Karyotype/subtelomeric test/BAC aCGH	17q12 (32,224,021–33,288,139)x3	*HNF1B*	1.1	FISH/–	unknown	Moderate ID, pre- and postnatal microcephaly, length asymmetry of limbs: left tibial and foot hypoplasia; dysmorphic features: round, distinctive face, short palpebral fissures, up-slanting palpebral fissures, and micrognathia; family history: two sisters and one brother: mild ID, father: mild ID, brother of proband’s father and his son: mild ID, proband’s grandfather: mild ID and two cases of preterm birth (babies died soon after birth)	614526
33	M	10	Karyotype/subtelomeric test/FraX	17q21.31 (41,153,459–41,494,390)x1	*KANSL1*	0.34	FISH/FISH	de novo	Delayed psychomotor development, moderate ID, and feeding problems in the neonatal/infancy period; dysmorphic features: frontal bossing, long face, abnormal philtrum, malformed auricles, low-set ears, and broad nasal bridge, family history: paternal uncle: case of newborn death (congenital abnormalities)	610443
34	F	1	Karyotype/subtelomeric test/microdeletion test	17q24.2 (62,016,186–63,909,858)x1	*PRKCA*	1.9	FISH/FISH	de novo	Supplementary material	(Vergult et al. 2012)
35	M	11	Karyotype	19p13.3 (1,083,903–3,636,080)x3	*STK11*	2.5	FISH/aCGH	de novo	Moderate ID, feeding problems, poor weight gain, IUGR and postnatal microcephaly and short stature; dysmorphic features: up-slanting palpebral fissures, hypertelorism, long nose, short philtrum, micrognathia, astigmatism, and camptodactyly	(Scollon et al. 2012)
36^e^	M	4	Karyotype/subtelomeric test	Xp22.31 (6,876,449–8,057,511)x0	*STS*, *VCX*	1.18	FISH/FISH	mat	Delayed psychomotor development, severe ID, absent speech, muscular hypotonia, postnatal microcephaly (−4.41 SD), clinodactyly of the 5th finger, inability to walk unsupported, epilepsy, and symptoms of mild ichthyosis; family history: mother: healthy, maternal father: ichthyosis (normal intellectual development), two maternal sisters: carriers of deletion Xp22.31	308100
37	F	11	Karyotype	Xp22.12 (20,096,103–21,653,164)x1	*RPS6KA3*	1.5	MLPA/MLPA	not mat	Delayed psychomotor development, moderate ID, speech delay, behavioral abnormalities (autoaggression, hyperactivity), failure to thrive, joint laxity, kyphoscoliosis, and brachydactyly; dysmorphic features: down-slanting palpebral fissures, hypertelorism, macrostomia, low and wide nasal bridge; Coffin–Lowry syndrome symptoms	303600
38	M	3	Karyotype/subtelomeric test	Xp11.22 (54,085,532–54,130,083)x0	*PHF8*	0.044	–/aCGH	mat	Delayed psychomotor development, speech delay, hearing loss, IUGR, neonatal feeding difficulties: poor suck, axial hypotonia, limbs hypertonia, postnatal microcephaly (−2.77 SD), clinodactyly of 3rd and 4th fingers, and cryptorchidism; dysmorphic features: acrocephaly, high forehead, hypotelorism, up-slanting palpebral fissures, long, deep philtrum, telecanthus, low-set ears, broad nasal bridge, and bulbous nasal tip; family history: cases of mild/moderate/severe ID in the relatives	300263

DD, developmental delay; ID, intellectual disability; SD, standard deviation; IQ, intelligence quotient; ASD, atrial septal defect; VSD, ventricular septal defect; PDA, patent ductus arteriosus; PFO, patent foramen ovale; IUGR, intrauterine growth restriction; MRI, magnetic resonance imaging; EEG, electroencephalography; mat, maternal; pat, paternal

^a^Patient 1 has an additional 0.250-Mb duplication at 15q11.2 [15q11.2 (20,393,584–20,642,621)x3]

^b^Patient 19 has an additional 1.2-Mb duplication at Xp22.31 inherited from the normal mother [Xp22.31 (6,876,449–8,075,153)x2 mat]

^c^Patient 26 has an additional 0.07-Mb duplication at Xp22.33 [Xp22.33 (508,271–579,292)x2]

^d^Patient 30 has an additional 0.85-Mb duplication at 6q25.3 [6q25.3 (157,243,724–158,094,366)x3 dn]

^e^Patient 36 has an additional 0.14-Mb duplication at 17q25.3 inherited from the normal mother [17q25.3 (78,458,509–78,599,991)x3 mat]

The second group consists of 15 CNVs potentially pathogenic for DD/ID (Table 2). In three cases, we identified rare CNVs: two de novo deletions at 13q12.11 and 5q35.3, and in one patient, a deletion at 10q21.3 and a duplication at 19p13.42p13.43.

**Table 2:** CNVs potentially pathogenic for DD/ID

Pt	Sex	Age (years)	Previous negative genetic tests	aCGH results (hg 18)	Genes	Size (Mb)	Verification/parental studies	Inheritance	Clinical features
39	F	15	Karyotype/subtelomeric test	2p21 (45,248,256–45,793,855)x3 2q37.2q37.3 (236,606,748–238,019,834)x3	*SRBD1*, *PRKCE* *AGAP1*, *GBX2*, *ASB18*, *IQCA1*, *CXCR7*, *COPS8*, *COL6A3*	0.54 1.4	–/aCGH –/aCGH	pat mat	Profound ID, absent speech, neonatal feeding problems: postnatal failure to thrive, muscular hypotonia, epilepsy, abnormal brain MRI: frontal cortical atrophy, EEG: hypsarrhythmia, postnatal microcephaly (−3.84 SD), and scoliosis; dysmorphic features: dolichocephaly, long face, wide-spaced teeth, hypoplastic nails, and cafe-au-lait spots
40	M	9	Karyotype/FISH (22q11.2)	2q11.2q12.1 (100,896,293–104,521,737)x3	*NPAS2*, *RPL31*, *TBC1D8*, *C2orf29*, *SNORD89*, *RNF149*, *CREG2*, *RFX8*, *MAP4K4*, *IL1R2*, *IL1R1*, *IL1RL2*, *IL1RL1*, *IL18R1*, *IL18RAP*, *SLC9A4*, *SLC9A2*, *MFSD9*, *TMEM182*, *LOC150568*	3.6	–/–	unknown	Borderline intellectual development, autistic-like spectrum symptoms: Asperger syndrome, hyperactivity, cleft of soft palate: infancy feeding difficulties, and horseshoe kidney; dysmorphic features: up-slanting palpebral fissures, high palate, hypertelorism dysplastic ears, protruding ears, preauricular dimples, and broad and flat nasal bridge
41	F	7	Karyotype	3q26.1q26.2 (169,023,234–169,652,178)x3	*SERPINI1*, *GOLIM4*	0.62	–/–	unknown	Profound ID, absent speech, behavior abnormalities (aggression, hyperactivity), severe postnatal failure to thrive; dysmorphic features: down-slanting palpebral fissures, hypertelorism, low forehead, and hirsutism
42	F	11	Karyotype	3q26.31 (176,602,337–177,202,589)x1	*NAALADL2*	0.6	FISH/FISH	pat	Mild ID, bilateral optic nerve atrophy, unilateral aniridia, secondary glaucoma, unilateral cataract, nystagmus. absent septum pellucidum, and pes planus; dysmorphic features: prominent forehead, tapering fingers, and brachydactyly
43	F	3	Karyotype/FISH (7q11.23)	4p13p12 (44,346,805–45,850,451)x3	*YIPF7*, *GUF1*, *GNPDA2*, *GABRG1*	1.5	FISH/FISH	mat	Delayed psychomotor development, speech delay (only single words), neonatal feeding problems, poor weight gain, gastroesophageal reflux disease, recurrent urinary infections, relative postnatal macrocephaly postnatal short stature (−6.64 SD), and postnatal underweight (−3.71 SD); dysmorphic features: long, deep philtrum, coarse face; exotropia, barrel-shaped chest, accessory spleen, fetal finger pads, and molecular confirmation of mutation in the *BRAF* gene; clinical diagnosis of CFC syndrome
44^a^	F	5	Karyotype	5q23.1 (115,661,713–117,144,119)x3	*SEMA6A*	1.4	–/–	unknown	Severe ID, absent speech, epilepsy, and stereotyped movements; dysmorphic features: long, smooth philtrum, low nasal bridge, bulbous nasal tip, and prominent alae nasi
45	F	17	Subtelomeric test	5q35.3 (176,820,265–177,542,421)x1	***DBN1***, *PDLIM7*, *DOK3*, *DDX41*, *FAM193B*, *TMED9*, ***B4GALT7***, *LOC202181*, *FAM153A*, *LOC728554*, ***PROP1***, *FAM153C*, *N4BP3*, *RMND5B*, ***NHP2***	0.72	FISH/FISH	de novo	Supplementary material
46	M	15	Karyotype/FraX	7p22.1 (4,813,509–4,971,674)x1 7q21.11 (81,426,300–81,452,221)x1	*RADIL*, *PAPOLB*, *MMD2* *CACNA2D1*	0.15 0.025	–/– –/–	unknown unknown	Mild ID and Asperger syndrome symptoms; mild dysmorphic features: coarse face, protruding ears, brachydactyly, prominent interphalangeal joints, mild interdigital webbing, hallux valgus, and scrotum anomaly
47^b^	M	5	Karyotype/FraX	7p22.1 (5,501,609–5,802,469)x3	*FBXL18*, *MIR589*, *ACTB*, *FSCN1*, *RNF216*	0.3	–/aCGH	de novo	Delayed psychomotor development, severe ID, absent speech, behavioral abnormalities (aggression, hyperactivity), and joint laxity; dysmorphic features: dolichocephaly, shallow orbits, blue sclerae, anteverted nares, long, flat philtrum, protruding ears, and club foot
48	M	10	Karyotype/subtelomeric test/BAC aCGH	7q35q36.1 (146,565,422–148,102,720)x3	*CNTNAP2*, *MIR548F4*, *C7orf33*, *CUL1*	1.5	FISH/FISH	mat	Profound ID, absent speech, muscular hypertonia, infantile feeding difficulties, congenital microcephaly, postnatal microcephaly (−7.22 SD), postnatal failure to thrive, epilepsy, and abnormal brain MRI (corpus callosum hypoplasia); mild dysmorphic features: astigmatism, optic disc hypoplasia, and micropenis
49	F	12	Karyotype	8q22.1 (97,401,668–98,506,950)x3	*PTDSS1*, *SDC2*, *PGCP*, *TSPYL5*	1.1	–/–	unknown	Delayed psychomotor development, delayed speech, mild ID (learning difficulties), behavioral abnormalities (hyperactivity, aggression), peripheral neuropathy, cardiac abnormalities (mitral valve regurgitation), unilateral hearing loss, nasal voice, premature sexual maturation, astigmatism, hypermetropia, pes cavus deformity, dry skin, patchy hypopigmented and hyperpigmented lesions; dysmorphic features: plagiocephaly, facial symmetry, low-set ears, narrow palpebral fissures, and bulbous nose
50^c^	M	17	Karyotype/subtelomeric test/BAC aCGH	10q21.3 (68,965,664–70,334,377)x1	***CTNNA3***, ***DNAJC12***, ***SIRT1***, ***HERC4***, ***MYPN***, *ATOH7*, ***PBLD***, ***HNRNPH3***, ***RUFY2***, ***DNA2***, ***SLC25A16***, ***TET1***, ***CCAR1***, *SNORD98*, ***STOX1***, ***DDX50***	1.36	FISH/FISH	not mat	Supplementary material
51	M	3	Karyotype	13q12.11 (19,168,788–19,420,048)x1	***PSPC1***, ***ZMYM5***	0.25	FISH/FISH	de novo	Supplementary material

DD, developmental delay; ID, intellectual disability; SD, standard deviation; MRI, magnetic resonance imaging; EEG, electroencephalography; mat, maternal; pat, paternal

**Boldface** indicates the best candidate genes

^a^Patient 44 has an additional 0.18-Mb deletion at 4p16.3 [4p16.3 (175,365–358,881)x1 mat] inherited from the normal mother

^b^Patient 47 has an additional 0.2-Mb duplication at 2p25.3 and 0.25-Mb duplication at 15q11.2, both inherited from the normal mother [2p25.3 (56,097–261,916)x3 mat, 15q11.2 (20,393,584–20,642,621)x3 mat]

^c^Patient 50 had an additional 0.4-Mb duplication at 19q13.42q13.43 [19q13.42q13.43 (61,141,677–61,543,411)x3]

In the third group, we classified 28 deletions and duplications of unknown clinical significance, some of which are likely non-pathogenic for DD/ID (Tables 1, 2, and 3). We found four individuals with BP1/BP2 duplication at 15q11.2 that is frequently found in the general population and considered as a benign event.

**Table 3:** CNVs of unknown clinical significance (likely non-pathogenic) for DD/ID

Pt	Sex	Age (years)	Previous negative genetic tests	aCGH results (hg 18)	Size (Mb)	Verification/parental studies	Inheritance	Clinical features
52	M	14	Karyotype	1q21.1 (144,124,745–144,452,014)x3	0.327	–/–	unknown	Moderate ID, speech delay, articulation defects, learning difficulties, and EEG abnormalities; dysmorphic features: hypertelorism, large ears, protruding ears, deep philtrum, high forehead, ptosis, prognathism, feet abnormalities, partial cutaneous syndactyly of fingers 2 and 3, and obesity since early childhood
53	F	7	Karyotype	5q14.2 (82,442,763–82,552,656)x1	0.109	FISH/FISH	mat	Moderate ID and behavioral abnormalities; dysmorphic features: high forehead, bilateral epicanthus, dysplastic, prominent and low-set ears with up-lift ear lobule, broad nasal bridge, thick helix, upturned nasal tip, anteverted nares, prognathism, strabismus deep set eyes, and clinodactyly of Vth digits
54	M	7	Karyotype	7p15.3 (23,684,650–23,791,776)x1	0.107	FISH/FISH	pat	Delayed psychomotor development, moderate ID, speech delay, hyperactivity, and learning difficulties; dysmorphic features: up-slanting palpebral fissures, high palate, cleft soft palate, strabismus, neonatal feeding difficulties, and scoliosis; suspicion of Raynaud’s phenomenon
55^a^	M	7	Karyotype/FISH (15q11.2)	9p24.3 (1,699,690–3,026,335)x3	1.326	FISH/aCGH	pat	Mildly delayed motor development, moderate ID, significant delay in speech development, behavioral abnormalities with aggression, irritability, and short attention span, muscle hypotonia, extreme obesity, hypogenitalism, and ectropion of lower eyelids
56	M	1	Karyotype/methylation test	11p15.4 (4,428,038–4,810,618)x1	0.382	FISH/FISH	pat	Delayed psychomotor development, muscular hypotonia, epilepsy, postnatal short stature (−3.87 SD), postnatal underweight (−4.83 SD), Pierre Robin sequence, neonatal feeding difficulties, absent suck, neonatal recurrent vomiting; dysmorphic features: high forehead, down-slanting palpebral fissures, hypertelorism, low-set ears, broad nasal bridge, strabismus, and club feet; family history: father: healthy
57	F	11	FISH (22q11.2)/BAC aCGH	12p13.33 (1,738,901–2,063,559)x3	0.324	FISH/aCGH	de novo	Delayed psychomotor development, severe ID, postnatal short stature, cardiac abnormalities (ASD, VSD, PDA), severe sensorineural deafness hypothyroidism; dysmorphic features: facial asymmetry, hypotelorism, ptosis, long, hooked nose, cleft palate, micrognathia, neck webbing, radioulnar synostosis, myopia, accessory spleen, family history: 1× stillbirth without congenital malformations, 1× miscarriage (first trimester of gestation)
58	M	2	Karyotype	12q21.1 (70,966,697–71,222,382)x1	0.255	FISH/FISH	pat	Delayed psychomotor development, absent speech (inarticulate, single sounds), in the neonatal/infantile period of life: limbs hypertonia/axial hypotonia, seizures, pre-/postnatal microcephaly: (−5.65 SD), epilepsy, brain MRI: suspicion of septo-optic dysplasia, polymicrogyria, schizencephaly, and subependymal heterotopy; dysmorphic features: down-slanting palpebral fissures, hypotelorism, EEG abnormalities, genitourinary abnormalities: extrarenal pelvis (left kidney) and optic disc hypoplasia
59	M	8	Karyotype/subtelomeric test	13q32.1q32.2 (96,900,637–97,686,612)x3	0.785	FISH/aCGH	mat	Delayed psychomotor development, profound ID, absent speech, neonatal and infantile feeding problems, muscular limbs hypertonia, seizures, drug-resistant epilepsy, pre- and postnatal microcephaly, stereotyped movements; dysmorphic features brachycephaly, micrognathia, protruding ears, open mouth appearance, full cheeks, abnormal brain MRI: temporal cortical atrophy (right), scoliosis, cryptorchidism, nystagmus, hematologic abnormalities, and unexplained transient highly elevated leukocytosis (30,000/ml); family history: proband’s sister: very similar clinical symptoms suggestive for unexplained encephalopathy with drug-resistant epilepsy
60	M	15	Karyotype	15q11.2 (19,924,765–20,642,621)x3	0.717	–/–	unknown	Delayed psychomotor development, profound ID, absent speech, stereotyped movements, neonatal/infantile feeding difficulties, poor suck, poor weight gain, postnatal failure to thrive, pectus excavatum, chondro-osseous exostoses on the lower limbs, strabismus, and secondary osteoporosis (hypercalciuria)
61	M	10	Karyotype	15q11.2 (20,006,102–20,642,621)x3	0.636	–/–	unknown	Delayed psychomotor development, moderate ID, speech delay, articulation defect, learning difficulties, vertebral and rib anomalies, short neck, postnatal short stature (relatively short lower limbs), postnatal underweight, heart defect (ASD, VSD), and strabismus; family history: 1× miscarriage in the mothers’ gestational history; features of Jarcho–Levin-like syndrome
62	M	6	Subtelomeric test	15q21.3 (54,693,214–54,832,701)x1	0.139	FISH/FISH	mat	Delayed psychomotor development, moderate ID, neonatal/infantile feeding difficulties, joints laxity, pectus excavatus, strabismus, and heart defect (VSD); dysmorphic features: Kabuki-like makeup syndrome, ectropion of eyelids, wide palpebral fissures, epicanthic folds, prominent ears, flat philtrum, and fetal finger pads
63	F	14	Karyotype/FraX	16q23.1 (77,445,915–78,190,209)x3	0.744	–/aCGH	not mat	Mild/moderate ID, mild dysmorphic features, hypertelorism, high forehead transverse palmar crease, and low posterior hairline
64	F	6	Karyotype/BAC aCGH/methylation test	22q11.21q11.22 (20,269,922–21,393,710)x3	1.123	–/–	unknown	Severe ID, absent speech, stereotyped movements, breath holding, postnatal microcephaly, inability to walk independently, rash of upper limbs; dysmorphic features, short philtrum, uplift earlobes, thick and sparse eyebrows, flat facial profile, protruding tongue, exotropia, and gum hypertrophy; family history: 1× miscarriage; clinical diagnosis of Rett syndrome, molecular confirmation of *MECP2* mutation
65	M	11	Karyotype/subtelomeric test	Xp22.33 (263,265–690,981)x3	0.427	–/–	unknown	Mild ID, speech delay, Postnatal microcephaly, abnormal brain MRI: arachnoid cyst and cerebellar vermis hypoplasia, EEG abnormalities, heart defect: tetralogy of Fallot (TOF), unilateral inguinal hernia, and vesicoureteral reflux; dysmorphic features: hypertelorism, telecanthus, short philtrum, downturned corners of the mouth, and thin lips
66	F	4	–	Xp22 (502,226–1,744,236)x3	1.242	–/–	unknown	Delayed psychomotor development, severe ID, speech delay (only single words), and autistic spectrum behavior; dysmorphic features: almond-shaped eyes, short philtrum, downturned corners of the mouth, prominent nasal tip, and proximal placement of the thumbs; family history: 1× miscarriage in the mother
67	F	18	Karyotype/subtelomeric test/FraX/FISH(1p36)	Xq27.3 (142,571,903–142,766,693)x3	0.194	–/aCGH	not mat	Profound ID and absent speech (only single words); dysmorphic features: deep-set eyes, straight supraorbital ridges, postnatal microcephaly, behavior abnormalities: aggression, autoaggression, stereotyped movements, obesity, and small hands and feet; family history: mother: mild ID
68	F	2	Karyotype/subtelomeric test/HR-CGH	Xq28 (151,572,279–151,788,528)x3	0.216	–/aCGH	pat	Delayed psychomotor development, speech delay (only single words), relative postnatal microcephaly (−2.05 SD), feeding difficulties, absent suck, distal arthrogryposis, (dimples over the interphalangeal joints), and premature thelarche; dysmorphic features: up-slanting palpebral fissures, long philtrum, downturned corners of the mouth, metopic ridge, and strabismus
69	M	15	Karyotype/subtelomeric test/FraX	Xq28 (152,641,149–152,697,974)x3	0.056	–/–	unknown	Profound ID, absent speech, behavioral abnormalities (hyperactivity, aggression), autism spectrum symptoms, muscular hypotonia, microcephaly, epilepsy, brachydactyly, alopecia areata, thin skin with hyperpigmentation in the perioral and periorbital regions; family history: stillbirth newborn from the mothers’ first pregnancy died after birth (IUGR without congenital malformations) and 2× miscarriages at the first trimester of the mother’s pregnancies

DD, developmental delay; ID, intellectual disability; SD, standard deviation; ASD, atrial septal defect; VSD, ventricular septal defect; PDA, patent ductus arteriosus; IUGR, intrauterine growth restriction; MRI, magnetic resonance imaging; EEG, electroencephalography; mat, maternal; pat, paternal

The total number of CNVs of unknown clinical significance includes CNVs in Table 3 and the additional changes listed under Tables 1, 2, and 3

^a^Patient 55 with a karyotype 46,XY,t(2;11)(q21;q23) has an additional 0.754-Mb deletion at 2q22.11 inherited from the normal mother [2q22.1 (138,045,798–138,799,860)x1 mat]

Moreover, we present the cumulative data of the studied cohort (Table 4), including patients with normal array CGH results (Supplementary Table 1).

**Table 4:** Summary of the studied cohort of 256 patients with DD/ID

Patients with	Total number (256)	Number of patients with CNVs (69)	Reported CNVs			Number of patients with two CNVs
			Pathogenic (41)	Potentially pathogenic (15)	Variants of unknown significance (28)	
DD (age 1–6 years)	74	17 (22.9 %)	12 (16.2 %)	2	4	1
Mild ID	32	11 (34.3 %)	6 (18.7 %)	5	2	2
Age <12 years	22	7	4	3	1	–
Age 13–18 years	8	4	2	2	1	–
Age >18 years	2	–	–	–	–	–
Moderate ID	57	18 (31.5 %)	11 (19.2 %)	2	10	5
Age <12 years	35	13	8	–	8	–
Age 13–18 years	20	4	2	2	2	–
Age >18 years	2	1	1	–	–	–
Severe ID	32	10 (31.2 %)	5 (15.6 %)	2	8	4
Age <12 years	25	9	4	2	8	–
Age 13–18 years	7	1	1	–	–	–
Age >18 years	–	–	–	–	–	–
Profound ID	61	13 (21.3 %)	7 (11.4 %)	4	4	2
Age <12 years	35	7	5	2	1	–
Age 13–18 years	19	6	2	2	3	–
Age >18 years	7	–	–	–	–	–

## Discussion

To determine the phenotypic consequences of the 84 identified non-polymorphic CNVs, we considered their type (deletion vs. duplication), size, gene content, inheritance pattern, and the available clinical or genomic database information. We classified them into three groups: 41 (16.0 %) known well-recognized causative for DD/ID, 15 (5.85 %) novel potentially pathogenic, and 28 (10.9 %) variants of unknown clinical significance (Tables 1, 2, and 3). The first group includes CNVs responsible for the well-known diseases and syndromes or published genomic imbalances that were considered as clinically relevant for DD/ID. The second group consists of novel CNVs potentially causative for DD/ID and includes changes that were not previously reported to be associated with DD/ID but contain genes that may contribute to our patients’ phenotypes. Moreover, we compared CNVs classified as the second group with the publically available databases. The same or similar sized aberrations were not found in the Database of Genomic Variants (DGV, http://projects.tcag.ca/variation/), Database of Chromosomal Imbalance and Phenotype in Humans Using Ensembl Resources (DECIPHER, http://decipher.sanger.ac.uk), or the International Standards for Cytogenomic Arrays database (ISCA, https://www.iscaconsortium.org). In the third group of variants of unknown clinical significance, we classified CNVs that contain genes that were not previously associated with DD/ID, are frequently found in the general population, or were inherited from a healthy parent.

From the second group of potentially pathogenic CNVs, we selected for the detailed description three novel rare, probably pathogenic, deletions containing candidate genes that may contribute to the abnormal phenotypes observed in our patients. Furthermore, in two of those cases (pt 51 and pt 45), deletions arose de novo that support their potential pathogenicity.

**Fig. 1:**
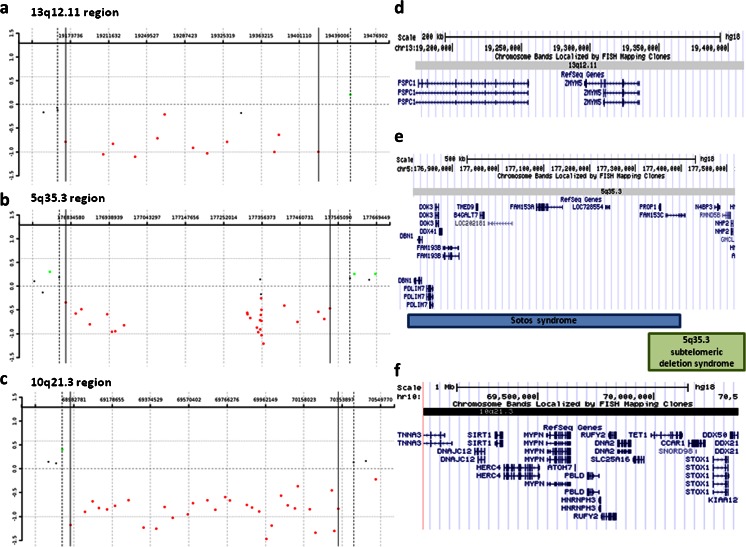
Results of array CGH analyses: **a** patient 51, showing a de novo ∼250-kb deletion in 13q12.11; **b** patient 45, demonstrating a de novo ∼720-kb deletion at 5q35.3; and **c** patient 50, showing a rare ∼1.4-Mb deletion in 10q21.3. The *red dots* denote the deleted region. Gene content in the deleted region on: **d** chromosome 13q12.11; **e** chromosome 5q35.3 (compared with the critical region of the Sotos syndrome and 5q35.3 subtelomeric deletion syndrome); and **f** chromosome 10q21.3 (UCSC genome browser, http://genome.ucsc.edu/)

A de novo ∼250-kb deletion in 13q12.11, encompassing only two genes, *PSPC1* (paraspeckle component 1; MIM 612408) and *ZMYM5* (zinc finger, MYM-type 5), was identified in pt 51 with remarkably delayed psychomotor development, muscle hypotonia, unilateral microphtalmia with ptosis, congenital eye malformation, and facial dysmorphic features (Fig. 1a, d). Interstitial deletions of 13q12.11 are very rare (Der Kaloustian et al. 2011; Tanteles et al. 2011). Tanteles et al. (2011) described a de novo 2.9-Mb 13q12.11 deletion of 19 genes, including *PSPC1* and *ZMYM5*, in a patient with near normal development and intellect but with scaphocephaly and facial dysmorphism. *PSPC1* encodes a nucleolar protein that localizes to punctate subnuclear structures close to splicing speckles, known as paraspeckles. Paraspeckles may function in the control of gene expression via an RNA nuclear retention mechanism. Functionally, *PSPC1* was proposed to participate in the regulation of transcription (Passon et al. 2011). ZMYM5 belongs to the zinc finger MYM-type family and probably has molecular functions, such as metal ion binding or zinc ion binding (Pastorcic and Das 2007). Although the phenotype–genotype correlation remains unclear and one smaller deletion within this region was reported in the control group (DGV; nsv455826) (Itsara et al. 2009), we propose that the identified de novo 13q12.11 deletion could contribute to the clinical features observed in our patient.

In a girl (pt 45) with an atypical autism spectrum disorder and moderate mental impairment with absent expressive speech development, we detected a de novo ∼720-kb deletion at 5q35.3 that partially overlaps a common-sized ∼1.9-Mb recurrent deletion found in patients with Sotos syndrome (MIM 117550) but leaves the dosage-sensitive *NSD1* gene (set domain protein 1; MIM 606681) intact (Fig. 1b, e). Rauch et al. (2003) described a novel 5q35.3 subtelomeric deletion distally adjacent to the Sotos common deletion region, and characterized by pronounced muscular hypotonia, postnatal short stature, and bell-shaped thorax with pectus carinatum. The deletion identified in our patient harbors 15 genes, including *DBN1*, *B4GALT7*, *PROP1*, and *NHP2*. *DBN1* (drebrin E; MIM 126660) is a cytoplasmic actin-binding protein thought to play a role in neuronal growth and dendritic spine formation. It is a member of the drebrin family of proteins that are developmentally regulated in the brain. Shim and Lubec (2002) and Dun and Chilton (2010) suggested that a decreased amount of drebrin may lead to loss of spine plasticity and impaired dendritic arborization, which may underlie cognitive dysfunction. PROP1 (paired-like homeodomain transcription factor; MIM 601538) has both DNA-binding and transcriptional activation ability. Its expression leads to ontogenesis of pituitary gonadotropes, as well as somatotropes, lactotropes, and caudomedial thyrotropes. Heterozygous mutations in *PROP1* have been reported in patients with pituitary hormone deficiency-2 (CPHD2; MIM 262600). Homozygous mutations of *B4GALT7* (galactosyltransferase I; MIM 604327) have been described in patients with the progeroid form of Ehlers–Danlos syndrome (MIM 130070) characterized by an aged appearance, DD, short stature, craniofacial disproportion, generalized osteopenia, defective wound healing, hypermobile joints, hypotonic muscles, and loose but elastic skin (Okajima et al. 1999). Lastly, homozygous or compound heterozygous mutations in the *NHP2* gene (nucleolar protein family A, member 2; MIM 606470) have been found in individuals with autosomal recessive dyskeratosis congenita-2 (DKCB2; MIM 613987). Given that the 5q35.3 deletion in our patient arose de novo and the fact that the deleted genes are associated with neurodevelopmental disorders and play a role in neuronal processes, we suggest that the identified CNV is potentially pathogenic for the described clinical features.

In patient 50 with short stature, moderate ID, and history of Hirschsprung disease and congenital heart defect, we found a rare ∼1.4-Mb deletion in 10q21.3 encompassing 16 genes (Fig. 1c, f) and a small ∼400-kb duplication in 19q13.42q13.43. Deletions in 10q21.3 have never been reported in patients with DD/ID, thus, it is challenging to assess its clinical pathogenicity. However, the size and gene-rich content suggest that the 10q21.3 deletion could be pathogenic. Analysis of the maternal sample revealed normal result; unfortunately, the paternal sample was unavailable. Five genes, including *SIRT1* (sirtuin 1; MIM 604479), *DNA2* (DNA replication helicase 2; MIM 601810), *TET1* (Tet oncogene family, member 1; MIM 607790), *CCAR1* (cell division cycle and apoptosis regulator 1; MIM 612569), and *DDX50* (dead/h box 50; MIM 610373) represent dosage-sensitive genes (Huang et al. 2010); however, to date, no phenotype associated with haploinsufficiency of any of these genes has been reported. In addition, *SLC25A16* (solute carrier family 25, member 16; MIM 139080) encodes a protein that is localized in the inner membrane and facilitates the rapid transport and exchange of molecules between the cytosol and the mitochondrial matrix space. This gene was proposed to play a role in Graves disease (MIM 275000). Of note, other SLC family genes, *SLC9A9* (MIM 608396), *SLC6A4* (MIM 182138), and *SLC25A12* (MIM 603667), are causative for autism. Other genes mapping in the deleted region, including *DNAJC12* (MIM 606060), *HERC4* (MIM 609248), *PBLD* (MIM 612189), *HNRNPH3* (MIM 602324), *RUFY2* (MIM 610328), *STOX1* (MIM 609397), and *CTNNA3* (MIM 607667), have been linked to late-onset Alzheimer’s disease (AD6; MIM 605526). *CTNNA3*, encoding the alpha-3 catenin and likely responsible for the formation of stretch-resistant cell–cell adhesion complexes (Weiss et al. 2009), has been associated with late-onset Alzheimer’s disease in females (Miyashita et al. 2007). Bradley et al. (2010) suggested that, if *CTNNA3* is involved in Alzheimer’s disease, it is not through a loss-of-function mechanism. Moreover, we suggest that *CTNNA3* and/or *MYPN* (myopalladin, MIM 608517), deleted 10q21 region, can be responsible for the congenital heart defect observed in our patient. *CTNNA3* has been considered as a candidate for the form of dilated cardiomyopathy linked to 10q21q23 (CMD1C; 601493) because of its high expression in the heart. MYPN is a component of the sarcomere that tethers nebulette in cardiac muscle to alpha-actinin and may play signaling roles in targeting and orienting nebulin to the Z line during sarcomere assembly. The associated ∼400-kb duplication in 19q13.42q13.43 harbors seven genes (*NLRP8*; MIM 609659, *NLRP5*; MIM 609658, *ZNF787*, *ZNF444*; MIM 607874, *GALP*; MIM 611178, *ZSCAN5B*, and *ZSCAN5A*), none of which represent a dosage-sensitive gene. Therefore, we classified this duplication as a variant of unknown clinical significance.

Among our 41 CNVs known as being pathogenic for DD/ID, we elected to discuss two deletions, 4q21 and 17q24.2, which have been described recently as responsible for microdeletion syndromes associated with DD/ID. Moreover, in those patients (pt 23 and pt 34), previously performed genetic tests were negative (Table 1), which demonstrates the usefulness of array CGH as a great tool for identifying the etiology of idiopathic DD/ID.

A de novo ∼3.1-Mb deletion at 4q21.21q21.22 identified in patient 23 overlaps CNVs reported in the literature (Harada et al. 2002; Friedman et al. 2006; Bonnet et al. 2010; Lipska et al. 2011). Bonnet et al. (2010) defined a 1.37-Mb critical region containing five genes, *PRKG2* (MIM 601591), *RASGEF1B* (MIM 614532), *HNRNPD* (MIM 601324), *HNRPDL* (MIM 607137), and *ENOPH1*, and proposed that *PRKG2* and *RASGEF1B* are the best candidate genes responsible for the 4q21 deletion syndrome (MIM 613509) characterized by severe ID, lack of speech, hypotonia, significant growth restriction, and distinctive facial features. Our patient enables a better delineation of a novel 4q21 microdeletion syndrome and further supports the proposed contribution of haploinsufficiency of *PRKG2* and *RASGEF1B*.

We also identified a de novo ∼1.9-Mb deletion at 17q24.2 (pt 34), harboring the smallest region of overlap of four deletions recently reported by Vergult et al. (2012). The shared clinical features include ID, speech delay, truncal obesity, and similar facial gestalt. The ∼713-kb critical region contains five genes, including *PRKCA* (MIM 176960), a cluster of three *CACNG* genes encoding the gamma subunit of a voltage-dependent calcium channel, *CACNG5* (MIM 606405), *CACNG4* (MIM 606404), *CACNG1* (MIM 114209), and *HELZ* (MIM 606699). The *PRKCA* gene encodes the serine- and threonine-specific protein kinase C alpha that plays an important role in many different cellular processes and is the most important candidate gene for many of the observed clinical features. Investigations of more patients with 17q24.2 deletions would strengthen the genotype–phenotype correlation in this newly reported microdeletion syndrome.

Moreover, in two patients (pt 15 and pt 16) with normal standard cytogenetic results, we detected chromosomal aneuploidy in the form of a mosaic trisomy 9 using array CGH. In both cases, chromosomal mosaicism was confirmed by retrospective GTG banding analysis that revealed low-level mosaicism for trisomy 9 of 12.5 % and 8 %, respectively. Additionally, in patient 17 with DD and combined immunodeficiency and normal karyotype, we found a monosomy of chromosome 7 in his peripheral blood leukocytes. Chromosome 7 monosomy and 7q deletion have been frequently found in patients with myelodysplastic syndrome (MDS). MDSs are a heterogeneous group of stem cell disorders characterized by ineffective hematopoiesis, dysplastic changes in bone marrow and peripheral blood, and the risk of transformation to acute myeloid leukemia (AML) (Cordoba et al. 2012). We suggest that the identified aberration is responsible for the phenotypic abnormalities observed in our patient.

Additionally, our analyses of the DD/ID cohort revealed patients with more than one clinically relevant CNVs. Apart from patients 9, 10, and 12 with two pathogenic CNVs, we identified two patients (pt 39 and pt 46) with two rare potentially pathogenic changes. A complex clinical presentation in those patients supports a second-hit model, in which the compound effect of multiple CNVs, including those of unknown pathogenic significance, contributes to the phenotypic heterogeneity (Girirajan et al. 2012).

These results further demonstrate that array CGH, in addition to the identification of CNVs and chromosomal aneuploidies, also enables the detection and estimation of their low-level mosaicism that may remain undetected by conventional cytogenetic methods (Cheung et al. 2007). Furthermore, our results support the usefulness of array CGH in the identification of the aneuploidy of cells under-represented in the T-cell population missed by routine chromosome analysis. However, apart from balanced aberrations (e.g., translocations, inversions), array CGH without SNP oligonucleotides cannot detect uniparental disomy.

In summary, we found that 69 of 256 patients with DD/ID carry one or more CNVs, in 38 cases responsible for the observed clinical features and in 13 patients potentially pathogenic for DD/ID. Our results further confirm the usefulness of array CGH in the detection of pathogenic CNVs in patients with idiopathic neurodevelopmental disorders.

## Electronic supplementary material

Below is the link to the electronic supplementary material.

ESM 1 (DOC 106 kb)
