# Supplementary material for: Application of array comparative genomic hybridization in 256 patients with developmental delay or intellectual disability
Source: J Appl Genet. 2013 Dec 3;55(1):125–44. doi: 10.1007/s13353-013-0181-x (PMC3909616; doi:10.1007/s13353-013-0181-x)
Supplement: Supplementary file 1 — (DOC 106 kb) [file 13353_2013_181_MOESM1_ESM.doc]

**SUPPLEMENTARY MATERIAL**

**Application of Array Comparative Genomic Hybridization in 256 Patients**

**with Developmental Delay or Intellectual Disability**

Magdalena Bartnik^1^, Beata Nowakowska^1^, Katarzyna Derwińska^1^, Barbara Wiśniowiecka-Kowalnik^1^, Marta Kędzior^1^, Joanna Bernaciak^1^, Kamila Ziemkiewicz^1^, Tomasz Gambin^2^, Maciej Sykulski^3^, Natalia Bezniakow^1^, Lech Korniszewski,^4^ Anna Kutkowska-Kaźmierczak^1^, Jakub Klapecki^1^, Krzysztof Szczałuba^1^, Chad A. Shaw^5^, Tadeusz Mazurczak^1^, Anna Gambin^6^, Ewa Obersztyn^1^, Ewa Bocian^1^, Paweł Stankiewicz^1,5^

1) Department of Medical Genetics, Institute of Mother and Child, Warsaw, Poland; 2) Institute of Computer Science, Warsaw University of Technology, Warsaw, Poland; 3) Institute of Informatics, University of Warsaw, Warsaw, Poland; 4) Institute of Physiology and Pathology of Hearing, World Hearing Center, Genetic Counseling Unit, Warsaw, Poland 5) Department of Molecular & Human Genetics, Baylor College of Medicine, Houston TX, USA; 6) Mossakowski Medical Research Centre, Polish Academy of Sciences, Warsaw, Poland

**Patients**

**Patient 23** is a girl, born as the 2nd child of healthy and non-consanguineous parents. At the time of delivery, her mother and father were both 31-year-old. The pregnancy was uncomplicated and spontaneous delivery occurred at 37 week of gestation. Birth weight was 2.300 g (10-25 centile), body length 50cm (75-90 centile), and Occipito-Frontal Circumference (OFC) 32 cm (10-25 centile). Congenital pneumonia was diagnosed and treated after birth. Breast feeding was continued for one year without problems but tendency to excessive body weight was observed. At the age of 12 months, her weight was 10 kg (50 - 75 centile), body length 70 cm (3 centile), and OFC was 44.5 cm (10 - 25 centile). During 1st year of life, muscle hypotonia, nocturnal cry, anxiety, and psychomotor delay were noted. She could not sit unsupported before 12 months and was not able to walk without support before 28 months of life. At the age of 7 years 10 months, she articulates only few syllables and communicates by signs. Her hearing and eyesight are normal as well as the results of hormonal and biochemical investigations in the blood and urine. Although there was no history of seizures, abnormal electroencephalography with generalized changes were registered. Brain Magnetic Resonance Imaging (MRI) at the age of 5 years showed areas of dysmyelination/demyelination in white and grey matter of parieto-occipital regions of brain tissue. On physical examination, she was obese and had mild dysmorphic features with long eyelashes, thick eyebrows, thick lips, several round shape patchy depigmentation of the skin localized on the abdomen and lower limbs, and relatively small hands and feet.

**Patient 34** is the 3-year-old girl referred to Genetic Counseling Unit as a 10 months infant due to her dysmorphic features, hypotrophy, and congenital heart defect. She is the 3rd child of both 36-year–old healthy and non-consanguineous parents. Delivery was induced at 36 week of gestation by Cesarean section because of marked oligohydramnios, pelvical fetal position, and Intrauterine Growth Restriction (IUGR). Her birth weight was 1.780g (< 5 centile), body length 46 cm (25-50 centile), and OFC 29 cm (< 5 centile). Cardiac defect (Ventricular Septal Defect ,VSD) and unilateral hip dysplasia were diagnosed after birth. At the age of 3 months, she was hospitalized at the Pediatric Unit because of feeding difficulties with poor suck and poor body growth and unexplained fever. Submucous cleft palate was found and feeding by gastric tube was necessary for a few weeks. Antibiotic therapy because of Chlamydial pneumonia was also applied. At the age of 18 months, a marked hypotrophy was noted. Her body weight was 5.150 g < 3 centile), body length 66.5 cm <3 centile), and head circumference 41.0 cm (<3 centile)). Relatively enlarged abdominal and subscapular folds (> 75 centile) were present. There were no other structural body malformations but the following dysmorphic facial features were noted: sparse eyebrows, hypertelorism, high forehead, epicanthus, prominent dysplastic ears, down-turned corners of the mouth, thin upper lip, and 5th finger clinodactyly. Global DD became apparent from 10 months of life when she was unable to sit without support; and at the age 3 years, she could not stand up and walk. Her social development is relatively good and she interacts with others but her speech development is significantly delayed. She verbalized only few single words. Until 3 years of life, she had no skin abnormalities, behavioral problems, or seizures.

**Patient 45** was evaluated at the age of 16 years. She is the 2^nd^ child born spontaneously at 39 weeks of gestation to non-consanguineous healthy parents after uncomplicated pregnancy. At birth, the infant had growth retardation with weight 2.450g (< 5 centile), length 48 cm (25 centile) and head circumference 31 cm (< 5 centile). Her motor development was delayed; she sat at 12 months and walked at 24 months of life. In childhood, she had behavioral abnormalities with anxiety and crying. Atypical autism spectrum disorders and moderate mental impairment with absent expressive speech development were diagnosed. Since 8 years of life, she has started to manifest first symptoms of a bipolar disorder. There were no abnormalities in brain MRI, biochemical, or hormonal investigations. On physical examination at the age of 16 years, short stature 147.6cm (<3 centile), body weight 65.5 kg (90 centile) and BMI 30.1 (>97 centile). Sparse hair on the scalp were noted, but there were no dysmorphic features or congenital structural anomalies.

**Patient 50** was evaluated at Genetic Counseling Unit at age of 14 years because of short stature, moderate ID, history of Hirschsprung disease and congenital heart defect. He is the 3rd child of healthy and non-consanguineous parents. There was no family history of previous miscarriages, congenital malformations, or mental impairment. He was born after uncomplicated pregnancy at 37 week of gestation with the following body parameters: weight 2.650g (50-75 centiles), length 50 cm (75-90 centiles), OFC 32 cm (10-25 centiles) and Apgar scores 9 points in 1st and 5th minute of life. Newborn jaundice with elevated levels of bilirubin in the blood (18.0 mg/dl) between 4 and 7 day of life was treated by phototherapy. Feeding difficulties with failure to thrive and hypotonia were noted in infancy. Alternating constipation and diarrhea were also noted from birth. At the age of 6 months, congenital heart defect (VSD and Patent Ductus Arteriosus, PDA) were diagnosed and repaired twice at 9 months and 2 years. His psychomotor development was delayed, he started sitting at 16 months and walking at the age 20 months. He had left inguinal hernia surgery at 10 years and successive surgery at 13 years because of Hirschsprung disease diagnosis. Anthropometric evaluation at the age of 14 years and 6 months showed short stature: 145.2 cm <3 centile) with relatively short upper and lower limbs (<3 centile). Comparison of his parents body parameters showed that his short stature is probably familial in origin (mother was 155 cm and father was 160 cm tall). On a physical examination, the following features were noted: strabismus, nystagmus, short philtrum, wide nasal bridge, large nose with bulbous tip, high palate, malocclusion with dysplastic malposition of anterior teeth, and brachydactyly. Result of a brain MRI showed focal perinatal ischaemic/anoxemic hypomyelination changes.

**Patient 51** is a male referred to genetic counseling unit at the age of 2 months because of congenital eye malformation and facial dysmorphic features. He is the 1st child of healthy and non-consanguineous parents. There was no family history of previous miscarriages, congenital malformations, or mental impairment. The pregnancy was complicated by mother’s arterial hypertension during second trimester of gestation and a single umbilical artery was identified in fetal ultrasonography. Spontaneous delivery occurred at 38 week of gestation. Birth weight of the boy was 2.810 g (10 centile), body length 54 cm (95 centile), and OFC was 33 cm (10 centile). Apgar scores were 10 points at 1st and 5th minute of life. At the age of 7 months, he demonstrated remarkably delayed psychomotor development with muscle hypotonia, unilateral microphtalmia with ptosis, choroidal coloboma, and nystagmus. He suffered from recurrent secretory otitis media and conductive hypoacusis was suspected. Persistent fetal pads, unilateral single palmar crease, scoliosis, mild facial dysmorphism, and hypoplastic nails were noted on physical examination. Developmental examination at 12 months of age showed global delay with skill levels of 6 months. Anthropometric evaluation at the age of 2 years and 8 months showed short stature, small body weight and OFC all parameters below 3rd centile. At this stage, his expressive speech development was severely delayed; he could verbalize only few simple words. He could not stand up and walk. There were no abnormalities in brain MRI evaluation.

Supplementary Table 1. Summary of patients with normal aCGH results.

| Patients with | Total Number | Number of patients and their age at diagnosis (y-years) | | | | | | | | | | | | | | | | | | | | | | |
| --- | --- | --- | --- | --- | --- | --- | --- | --- | --- | --- | --- | --- | --- | --- | --- | --- | --- | --- | --- | --- | --- | --- | --- | --- |
|  |  | 1y | 2y | 3y | 4y | 5y | 6y | 7y | 8y | 9y | 10y | 11y | 12y | 13y | 14y | 15y | 16y | 17y | 18y | 19y | 20y | 21y | 22y | 32y |
| **DD** | **57** | **7** | **16** | **10** | **12** | **5** | **7** | - | - | - | - | - | - | - | - | - | - | - | - | - | - | - | - | - |
| Males | 31 | 4 | 4 | 6 | 7 | 4 | 6 | - | - | - | - | - | - | - | - | - | - | - | - | - | - | - | - | - |
| Females | 26 | 3 | 12 | 4 | 5 | 1 | 1 | - | - | - | - | - | - | - | - | - | - | - | - | - | - | - | - | - |
| **Mild ID** | **21** | **-** | **-** | **-** | **-** | **1** | **-** | **2** | **4** | **1** | **2** | **3** | **2** | **-** | **-** | **1** | **2** | **-** | **1** | **1** | **1** | **-** | **-** | **-** |
| Males | 14 | - | - | - | - | - | - | 2 | 3 | 1 | 2 | 1 | 2 | - | - | - | 1 | - | 1 | - | 1 | - | - | - |
| Females | 7 | - | - | - | - | 1 | - | - | 1 | - | - | 2 | - | - | - | 1 | 1 | - | - | 1 | - | - | - | - |
| **Moderate ID** | **39** | **-** | **-** | **-** | **-** | **-** | **3** | **5** | **4** | **3** | **2** | **2** | **2** | **4** | **2** | **3** | **1** | **2** | **4** | **-** | **2** | **-** | **-** | **-** |
| Males | 27 | - | - | - | - | - | - | 4 | 4 | 3 | 2 | 2 | 2 | 3 | 2 | 2 | 1 | - | 2 | - | - | - | - | - |
| Females | 12 | - | - | - | - | - | 3 | 1 | - | - | - | - | - | 1 | - | 1 | - | 2 | 2 | - | 2 | - | - | - |
| **Severe ID** | **22** | **-** | **-** | **-** | **1** | **2** | **1** | **1** | **1** | **2** | **1** | **4** | **3** | **-** | **1** | **2** | **-** | **2** | **1** | **-** | **-** | **-** | **-** | **-** |
| Males | 14 | - | - | - | - | - | 1 | - | - | 2 | 1 | 4 | 2 | - | 1 | 1 | - | 1 | 1 | - | - | - | - | - |
| Females | 8 | - | - | - | 1 | 2 | - | 1 | 1 | - | - | - | 1 | - | - | 1 | - | 1 | - | - | - | - | - | - |
| **Profound ID** | **48** | **-** | **1** | **1** | **1** | **5** | **4** | **-** | **-** | **2** | **4** | **7** | **2** | **2** | **2** | **2** | **3** | **3** | **1** | **2** | **-** | **2** | **1** | **2** |
| Males | 29 | - | 1 | 1 | 1 | 3 | 4 | - | - | 1 | 1 | 6 | 1 | 2 | 1 | - | 2 | 1 | 1 | - | - | 1 | 1 | 1 |
| Females | 19 | - | - | - | - | 2 | - | - | - | 1 | 3 | 1 | 1 | - | 1 | 2 | 1 | 2 | - | 2 | - | 1 | - | 1 |
